# DEVELOPMENT AND ASSESSMENT OF AN INTERVENTION PROGRAM USING PAIR GO: RESULTS OF AN EXPLORATORY STUDY

**DOI:** 10.1093/geroni/igae098.3405

**Published:** 2024-12-31

**Authors:** Ai Iizuka, Kristine Mulhorn, Koki Ito, Moe Kitago, Chiaki Ura, Tsuyoshi Okamura, Kenji Toba, Hiroyuki Suzuki

**Affiliations:** Tokyo Metropolitan Institute for Geriatrics and Gerontology, Tokyo, Japan; Drexel University, Philadelphia, Pennsylvania, United States; Tokyo Metropolitan Institute for Geriatrics and Gerontology, Tokyo, Japan; Tokyo Metropolitan Institute for Geriatrics and Gerontology, Tokyo, Japan; Tokyo Metropolitan Institute for Geriatrics and Gerontology, Tokyo, Japan; Tokyo Metropolitan Institute for Geriatrics and Gerontology, Tokyo, Japan; Tokyo Metropolitan Institute for Geriatrics and Gerontology, Tokyo, Japan; Tokyo Metropolitan Institute for Geriatrics and Gerontology, Tokyo, Japan

## Abstract

The board game Go, a traditional board game that originated in Asia, is associated with positive effects on cognitive function in older adults. However, as a one-on-one game, Go usually requires a single winner and a single loser. Pair Go creates a team-based game where winning and losing is a shared experience. Pair Go enhances social interaction because it is played by pairs of players. Social interaction has a positive impact on the mental health of older adults. This study aimed to develop a new intervention program using Pair Go and to explore the impact on mental health and social interaction. This single-arm intervention study was conducted from October 2023 to January 2024 in Tokyo, Japan. Twenty-eight community-dwelling older adults (71-86 years) attended Pair Go classes once a week for 90 minutes, for 12 sessions. Each session involved attending lectures, solving exercises, and playing Pair Go. Mental health, and social interaction were assessed before and after the intervention. Comparison of pre- and post-test scores using paired t-tests revealed that scores on the University of California, Los Angeles Loneliness Scale were statistically significant lower in the post-test (mean [SD] score, 38.5 [6.6]) than the pre-test (36.6 [5.9]) (p<.05). In addition, 50% of the participants made new social connections in the Pair Go class, such as having tea or making phone calls after the program. These results indicate that the Pair Go program may affect mental health and enhance social interaction not seen with the original Go.

